# Viability and Antioxidant Effects of Traditional Cooling Rice Powder (*bedak sejuk*) Made from *Oryza sativa ssp. Indica *and *Oryza sativa ssp. japonica* on UVB-Induced B164A5 Melanoma Cells

**DOI:** 10.31557/APJCP.2020.21.11.3381

**Published:** 2020-11

**Authors:** Ahmad Rohi Ghazali, Raveena Vaidheswary Muralitharan, Chan Kam Soon, Tharsini Salyam, Nurul Najihah Ahmad Maulana, Ummul Aqeela Balqees Mohamed Thaha, Rasyidah Mohamad Halim, Sajidah Suhaifi, Muhamad Haziq Md Khalid, Adibah Hanis Ahmad, Noorhisham Tan Kofli

**Affiliations:** 1 *Programme of Biomedical Science, Centre of Applied and Health Sciences, Faculty of Health Sciences, Universiti Kebangsaan Malaysia, Kuala Lumpur, Malaysia. *; 2 *Department of Chemical and Process Engineering, Faculty of Engineering and Built Environment, Universiti Kebangsaan Malaysia, Bangi, Selangor, Malaysia.*

**Keywords:** Bedak sejuk, fermented rice, fermented cosmetics, Oryza sativa Indica, Oryza sativa Japonica

## Abstract

**Background:**

Traditional cooling rice powder (*bedak sejuk*) is a fermented rice-based cosmetic that is applied topically on one’s skin, as an overnight facial mask. According to user testimonies, *bedak sejuk *beautifies and whitens skin, whereby these benefits could be utilised as a potential melanoma chemopreventive agent.

**Objective:**

Hence, this study aimed to determine the effects of *bedak sejuk *made from *Oryza sativa ssp. indica (Indica)* and *Oryza sativa* *ssp. japonica (Japonica)* on UVB-induced B164A5 melanoma cells, and also identify the antioxidant capacities of both types of bedak sejuk.

**Methods:**

The optimum dose of *Indica* and *Japonica bedak sejuk *to treat the cells was determined via the MTT assay. Then, the antioxidant capacities of both types of *bedak sejuk *were determined using the FRAP assay.

**Results:**

From the MTT assay, it was found that *Indica* and *Japonica* *bedak sejuk *showed no cytotoxic effects towards the cells. Hence, no IC_50_ can be obtained and two of the higher doses, 50 and 100 g/L were chosen for treatment. In the FRAP assay, *Indica* *bedak sejuk *at 50 and 100 g/L showed FRAP values of 0.003 ± 0.001 μg AA (ascorbic acid)/g of *bedak sejuk *and 0.004 ± 0.0003 μg AA/g of bedak sejuk. Whereas *Japonica* *bedak sejuk *at 50 g/L had the same FRAP value as *Indica* *bedak sejuk *at 100 g/L. As for *Japonica* *bedak sejuk *at 100 g/L, it showed the highest antioxidant capacity with the FRAP value of 0.01 ± 0.0007 μg AA/g of *bedak sejuk *which was statistically significant (p < 0.05) when compared to other tested concentrations.

**Conclusion:**

In conclusion, *Japonica* *bedak sejuk *has a higher antioxidant capacity compared to *Indica* *bedak sejuk *despite both being not cytotoxic towards the cells. Regardless, further investigations need to be done before *bedak sejuk *could be developed as potential melanoma chemoprevention agents.

## Introduction

The largest organ of the human body is the skin, which accounts for 16 % of the human body weight. The epidermis of the skin functions as a protective layer that separates organisms from the external environment. This is crucial in counteracting environmental stressors such as ultraviolet (UV) light. When UV light penetrates the epidermis, the melanin pigments in keratinocytes form a protective cover above the keratinocytes nuclei. This confers protection for the skin against UV light penetration as well as neutralising reactive oxygen species (ROS) that have been produced (D’Orazio et al., 2013; Aluwi et al., 2016; Woolridge Cooper, 2018).

However, increased exposure towards UV light is the main cause of hyperpigmentation and skin damage (Chan et al., 2014; American Cancer Society, 2020). The UV light from the Sun that penetrates skin are UVA (90 - 95 %) and UVB rays (5 – 10 %), whereby the extent of skin damage is dependent on the wavelength of each ray. UVA rays have longer wavelengths (320 – 400 nm) that penetrate deep into the dermis and result in free radical formation, especially ROS. As for UVB rays, with shorter wavelengths (280 – 320 nm) that penetrate until the epidermal layer only, the rays damage DNA which in turn causes mutations. This combination of ROS formations and mutations ultimately contribute to the development of skin cancer (Sander et al., 2003; Pfeifer and Besaratinia, 2012; D’Orazio et al., 2013; Kamarulzaman et al., 2017; Pavel et al., 2017; Nagapan et al., 2018).

A type of skin cancer that arises due to overexposure of UV light is melanoma. Melanoma occurs when melanocytes grow out of control due to mutations in DNA. Although melanoma accounts for only less than 10 % of all skin cancers, it contributes to the majority of skin cancer related deaths. This can be attributed to melanoma’s high metastatic potential and resistance towards therapy (Pfeifer and Besaratinia, 2012; American Cancer Society, 2020). Therefore, melanoma chemoprevention strategies are more suitable in tackling this disease occurrence (Chhabra et al., 2017). 

In line with melanoma chemoprevention strategies, the idea to use a local cosmetic product that has the potential to decrease ROS generation and mutation on human skin, emerged. This product, traditional cooling rice powder, more commonly known as *bedak sejuk *in Malaysia, is a traditional fermented rice-based cosmetic product. Rice grains that have been fermented in previous studies to produce *bedak sejuk *are *Oryza sativa ssp. indica (Indica)* and *Oryza sativa ssp. japonica (Japonica) *(Dzulfakar et al., 2015a). *Indica* rice grains are long, flat, slender, shatter easily and have high amylose content while *Japonica* rice grains are short, round, do not shatter easily and have low amylose content (Ricepedia, n.d.). *Bedak sejuk *as a product of rice grain fermentation, is in the shape of water droplets or cone-shaped pastilles. When these pastilles are mixed with water and applied onto the skin as an overnight facial mask, it gives off a cooling effect. It has been supported by user testimonies from generation to generation, that claimed *bedak sejuk *is able to beautify as well as whiten skin (Dzulfakar et al., 2015b; Dzulfakar et al., 2016c; Johar et al., 2018). However, these testimonies have never been tested in the laboratory setting especially on its effects towards skin cells both normal and malignant. 

Hence, our study aimed to determine the effects of *bedak sejuk *made from the *Indica* and *Japonica* rice subspecies using UVB-induced B164A5 melanoma cells, as well as identifying the antioxidant capacities of both types of *bedak sejuk.*

## Materials and Methods

*Preparation of Indica and Japonica bedak sejuk*

The *bedak sejuk *made from the* Indica *and *Japonica *rice subspecies were kindly provided from the Faculty of Engineering and Built Environment, Universiti Kebangsaan Malaysia, 43600 Bangi, Selangor. In order to prepare *bedak sejuk, Indica* and *Japonica* rice grains were soaked in tap water at a ratio of 1:1 (w/v) in separate closed containers that were not sterilised. The rice grains were then allowed to undergo natural fermentation for 14 days at ambient temperature. On the 14^th^ day, the rice grains were filtered using a muslin cloth and soaked again in a new batch of water (w/v). The soaking of the rice grains was repeated six times for every 14 days, bringing the overall soaking process to 84 days. After 84 days, the resulting rice paste from each rice subspecies was collected and dried in an oven to produce the powdered form of *bedak sejuk *(Dzulfakar et al., 2016a). The *bedak sejuk *was then kept in a refrigerator at 4^o^C until further use. Prior to experiments, the *bedak sejuk *was dissolved in distilled water and filtered through a 0.22 µm Millipore syringe filter for sterilisation.

*Cell culture*

The B164A5 murine melanoma cell line was purchased from the European Collection of Authenticated Cell Cultures (ECACC). The cells were cultured in Dulbecco’s Modified Eagle Medium (DMEM) enriched with 10% foetal bovine serum (FBS), 1% Penicillin-Streptomycin mixture (Pen/Strep, 10,000 IU/ml), glucose and L-glutamine. The cells were then incubated in a humidified atmosphere at 37^o^C in 5% CO_2_. When the cell confluency reaches 80%, the cells were sub-cultured (Public Health England, n.d.). Before the assays were conducted, the growth curve of the B164A5 cells was plotted. From the growth curve, the doubling time of 24 hours was obtained, whereby this finding was supported by a study from Danciu et al., (2013).

*MTT assay*

The cytotoxicity of both types of *bedak sejuk *towards B164A5 cells were evaluated through the 3-(4,5-dimethylthiazol-2-yl)-2,5-diphenyl tetrazolium bromide (MTT) cell viability assay according to the method of Mosmann (1983) with slight modifications. 200 µL of 5 x 10^4^ cells were seeded in a 96-well flat-bottom plate and incubated for 24 hours in a humidified atmosphere at 37^o^C in 5% CO_2_. After 24 hours of incubation, the media was discarded and replaced with 200 µL of PBS. Then, the cells were exposed to UVB radiation at 30 mJ/cm^2^ for 36.4 seconds (Lin et al., 2002). Right after the UVB exposure, the PBS was discarded and the cells were treated with both types of *bedak sejuk *in serially diluted concentrations of 6.25, 12.5, 25, 50 and 100 g/L. As for the positive control, the cells were treated with menadione in serially diluted concentrations of 0.0625, 0.125, 0.25, 0.5 and 1 mM according to Basri et al., (2015), while the negative control was the untreated cells. Following treatment, the cells were incubated for another 24 hours. After the incubation period, 20 µL of 5 mg/mL MTT solution was added into each well and incubated for 4 hours. Then, 190 µL of mixture was discarded from each well and 200 µL of DMSO was added and incubated for 15 minutes. Finally, the plate was shaken for 5 minutes and absorbance readings were taken at 570 nm using a microplate reader. The percentage of cell viability was calculated using the following formula:

Cell viability (%)= (Mean OD of treated cells)/(Mean OD of negative control) ×100

*Ferric Reducing Antioxidant Power (FRAP) assay*

The antioxidant capacities of both types of *bedak sejuk *were determined via the FRAP assay as described by Benzie and Strain (1996). It is a method to evaluate the antioxidant power through the reduction of ferric (Fe^3+^) to ferrous ions (Fe^2+^) at a low pH that result in the formation of coloured ferrous-tripyridyltriazine complex. Firstly, the FRAP working reagent was prepared freshly by mixing acetate buffer (30 mM, pH 3.6), iron (III) chloride (FeCl_3_) solution (20 mM) and TPTZ solution (10 mM) in the ratio of 10:1:1. The prepared FRAP working reagent was then kept in a water bath at 37^o^C and protected from light. This is followed by the preparation of the iron (II) sulphate (FeSO_4_) calibration curve using serially diluted concentrations that range from 100 – 1,000 µM. Ascorbic acid was used as the positive control and had been prepared using serially diluted concentrations that range from 3.125 – 50 µg/mL, in the dark (Hasiah et al., 2011). As for the experimental steps, 50 µL of FeSO_4_, ascorbic acid and both types of *bedak sejuk *were added into their allocated wells in a 96-well plate. After that, 175 µL of warmed FRAP working reagent was added subsequently into each well. Then, the plate was incubated at 37^o^C for 5 minutes. Finally, the absorbance readings were taken at 595 nm using a microplate reader and the FRAP values were expressed as ascorbic acid equivalent antioxidant capacity (AEAC) (Gashahun and Solomon, 2019).

*Statistical analysis*

The SPSS v25 software was used for data presentation. Each test data from three independent experiments (n = 3) were expressed as mean ± SEM. One-way ANOVA test was used for comparison between means. Alpha used was 0.05 and p value < 0.05 was considered as statistically significant.

## Results

*Cytotoxicity of Indica and Japonica bedak sejuk*

**Figure 1:**
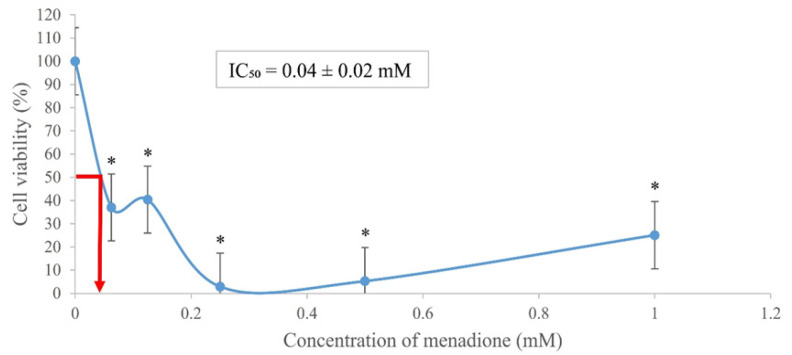
Cytotoxic Effect of Menadione on UVB-Induced B164A5 Melanoma Cell Viability at the Range of 0 – 1 mM Following 24 Hours of Treatment. Each concentration was compared to the negative control. * p < 0.05

**Figure 2:**
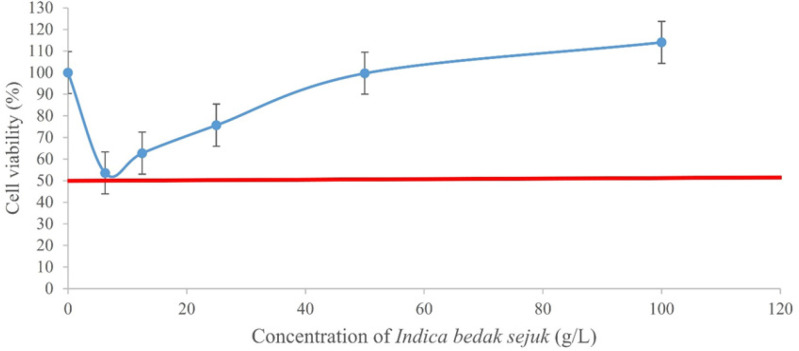
Effect of* Indica Bedak Sejuk* on UVB-Induced B164A5 Melanoma Cell Viability at the Range of 0 – 100 g/L Following 24 Hours of Treatment

**Figure 3:**
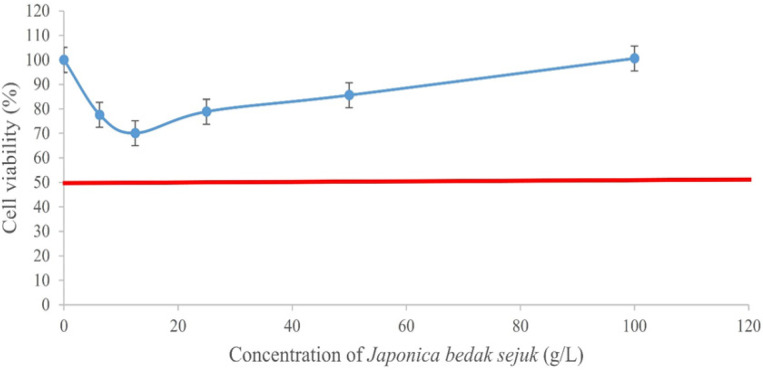
Effect of *Japonica bedak sejuk* on UVB-induced B164A5 Melanoma Cell Viability at the Range of 0 – 100 g/L Following 24 Hours of Treatment

The cytotoxicity of both types of *bedak sejuk *was evaluated against UVB-induced B164A5 melanoma cells via the MTT assay. Menadione, which was used as the positive control showed cytotoxicity with an IC_50_ of 0.04 ± 0.02 mM (Figure 1). Each concentration of menadione was statistically significant (p < 0.05) when compared with the negative control. Whereas *Indica *and* Japonica* *bedak sejuk *showed no cytotoxicity, hence no IC_50_ values were obtained. Both types of *bedak sejuk *had a reduction of cell viability at the beginning but gradually increased as the concentration increased (Figures 2 and 3). In addition, all concentrations of both types of *bedak sejuk *were not statistically significant (p > 0.05) when compared with the negative control. The results demonstrated that both types of *bedak sejuk *were not cytotoxic to UVB-induced B164A5 melanoma cells. 

*Antioxidant capacities of Indica and Japonica bedak sejuk*

**Figure 4:**
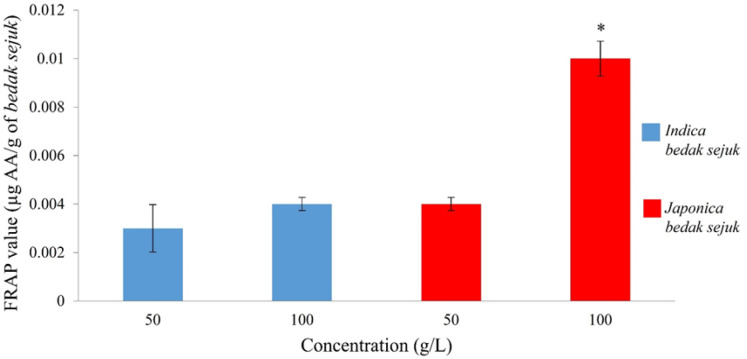
FRAP Values of Both Types of *bedak sejuk.* FRAP Value of *Japonica bedak sejuk* for 100 g/L was Significantly Higher than FRAP Values of *Japonica bedak sejuk* for 50 g/L and* Indica bedak sejuk* for 50 and 100 g/L. * p < 0.05

The reducing capabilities of both types of *bedak sejuk *as antioxidants were determined through the FRAP assay. The FRAP values were expressed as ascorbic acid equivalent antioxidant capacity (AEAC) in the unit µg AA (ascorbic acid)/g of *bedak sejuk*. The FRAP values for 50 and 100 g/L of *Indica bedak sejuk *were 0.003 ± 0.001 and 0.004 ± 0.0003 µg AA/g of *bedak sejuk *respectively (Figure 4). As for *Japonica bedak sejuk*, the FRAP values for 50 and 100 g/L were 0.004 ± 0.0003 and 0.01 ± 0.0007 µg AA/g of *bedak sejuk *respectively, showing a dose dependent manner (Figure 4). 

When the FRAP values of *Indica bedak sejuk *for 50 and 100 g/L were compared, it was not statistically significant (p = 0.803). But, the FRAP values of *Japonica bedak sejuk *for 50 and 100 g/L was statistically significant (p = 0.006) when compared. In addition, the FRAP value of *Japonica* *bedak sejuk *for 100 g/L showing the highest antioxidant capacity was statistically significantly when compared to FRAP values of *Indica bedak sejuk *for both 50 (p = 0.001) and 100 g/L (p = 0.003). 

## Discussion

Lately, the usage of natural products in skincare has been showing an increasing trend. Most of these natural products have been proven to have antioxidant characteristics in addition to providing protection to the skin against UV light (Abdul Wahab et al., 2014). One example of such a product is *bedak sejuk*, a fermented rice-based cosmetic product. For generations, *bedak sejuk *pastilles are mixed with water and applied topically on one’s skin as an overnight facial mask (Dzulfakar et al., 2016c; Johar et al., 2018).

In the fermentation of rice grains to produce bedak sejuk, lactic acid bacteria (LAB) are usually involved, alongside mould and yeast (Dzulfakar et al., 2015a; Dzulfakar et al., 2016b). The usage of cosmetic products that have this LAB fermented rice component results in the expansion and smoothness of the product as well as a wet feeling upon application on the skin (Sawaki et al., 2010). A similar experience is also reported by *bedak sejuk *users, whereby the application of *bedak sejuk *produces a cool feeling (Dzulfakar et al., 2015a). There is also an interesting relationship between the skin and the fermentation by LAB, whereby the LAB soaking water used during the fermentation of rice grains, contains lactic acid and other amino acids that contribute to skin hydration. These benefits make the LAB soaking water useful as a cosmetic product source. Hence, the combination of a substrate or medium like rice grains and the LAB strains may bring about cosmetic effects such as antioxidant effects, pH control and prevention of cell stress (Izawa and Sone, 2014). These benefits are in line with melanoma chemoprevention strategies. However, *bedak sejuk *has yet to be investigated for its effects on malignant skin cells in the laboratory. Hence, as a preliminary study for a potential melanoma chemoprevention agent, the B164A5 murine melanoma cells that have been UVB-induced is a suitable cancer model.

Firstly, in the MTT assay, it was found that* Indica *and *Japonica* *bedak sejuk *were not cytotoxic towards UVB-induced B164A5 melanoma cells. However, it was noted that there was a decrease in cell viability at lower concentrations of *bedak sejuk*, followed by an increase in cell viability at higher concentrations of *bedak sejuk. *This decrease in cell viability can be attributed to the fact that UVB rays are cytotoxic towards cells, in this case B164A5 cells (Pavel et al., 2017). At the same time, lower concentrations of *bedak sejuk *were not enough to increase the number of cells that have been UVB-induced. Regardless, the decrease in cell viability did not fall below the 50% mark and hence, no IC_50_ was able to be obtained for both types of *bedak sejuk.* As a result, two of the higher doses, 50 g/L and 100 g/L were chosen as the treatment doses for the next assay.

In the FRAP assay, it was found that *Japonica* *bedak sejuk *had a higher antioxidant capacity compared to that of *Indica bedak sejuk*. The differences could be explained by the fact that *Japonica *rice grains are cultivated in temperate and colder regions of Asia, while *Indica* rice grains are cultivated throughout tropical Asia (Garris et al., 2005). Environmental temperature plays an essential role in antioxidant activities of plants, and plants cultivated in colder weather have more pronounced antioxidant activities as opposed to plants that were cultivated in warmer weather. This increase in antioxidant activity can be attributed to the production of more phytochemicals as the plants undergo stress in colder weather (Kumar et al., 2017). 

There are also other fermented rice products that have exhibited promising results. Firstly, there is *Galactomyces *ferment filtrate (GFF) which is a byproduct of rice fermentation by the *Galactomyces *yeast. The GFF extract contains a unique blend of vitamins, minerals, small peptides and oligosaccharides that are used as cosmetic ingredients in skincare products. The extract has demonstrated antioxidant characteristics by protecting normal human epidermal melanocytes (NHEM) from oxidative stress (Woolridge Cooper, 2018). These findings of GFF was similar to the findings of *bedak sejuk *in this study.

Rice bran also possesses strong antioxidant activities to the point of resulting in cytotoxicity towards melanocytes. But, when the rice bran is fermented, the cytotoxicity of the rice bran extract towards B16F1 cells have been eliminated. This shows that the fermentation of rice brain produced new beneficial compounds with biological functions, although the exact compounds have yet to be elucidated (Chung et al., 2009). 

Finally, the rice soaking water from the powder of *bedak sejuk *actually contains amino acids that could be beneficial for cosmetic applications. There are 16 out of 17 amino acids detected in *Indica bedak sejuk *as well as its soaking water. Glutamic acid was the highest concentration of amino acid found in both *Indica bedak sejuk *and its soaking water (Johar et al., 2018). Glutamine (a glutamic acid derivative), alongside arginine, tyrosine and lysine that are detected in *bedak sejuk *and its soaking water are among the main amino acids that are used in cosmetic industries (Ha et al., 2018). Besides, amino acids in cosmetic products function as antioxidants (Ivanov et al., 2013) and the amino acid content in *Indica bedak sejuk *was much higher compared to its soaking water. Hence, the application of *bedak sejuk *was more effective in terms of amino acids content when compared to the soaking water (Johar et al., 2018).

In summary, *Indica* and *Japonica bedak sejuk *need to be investigated further before they can be said to have potential as a melanoma chemoprevention agent that is able to (1) prevent melanoma, (2) prevent development of malignant melanoma from pre-malignant lesions or (3) prevent reoccurrence of melanoma after successful melanoma treatment (Chhabra et al., 2017).
